# Incidentally detected tuberculous prostatitis with microabscess

**DOI:** 10.1590/S1677-5538.IBJU.2017.0190

**Published:** 2018

**Authors:** Eduardo Kaiser Ururahy Nunes Fonseca, Oskar Grau Kaufmann, Layra Ribeiro de Sousa Leão, Cassia Franco Tridente, Fernando Ide Yamauchi, Ronaldo Hueb Baroni

**Affiliations:** 1 Hospital Israelita Albert Einstein, São Paulo, Brasil

**Keywords:** Prostate, Tuberculosis, MRI, Radiology

## Abstract

Tuberculous prostatitis is a rare and often overlooked entity that may mimic prostatic adenocarcinoma on imaging exams, especially multiparametric magnetic resonance imaging (MRI) of the prostate. Detection of a prostatic abscess is a clue to the correct diagnosis.

## CASE PRESENTATION

A 73-year-old man with progressive lower urinary tract symptoms for five months was referred to our service for evaluation of prostatic enlargement. During this period, he was using indwelling bladder catheter. He denied any known epidemiological history or respiratory symptoms.

**Figure 1:**
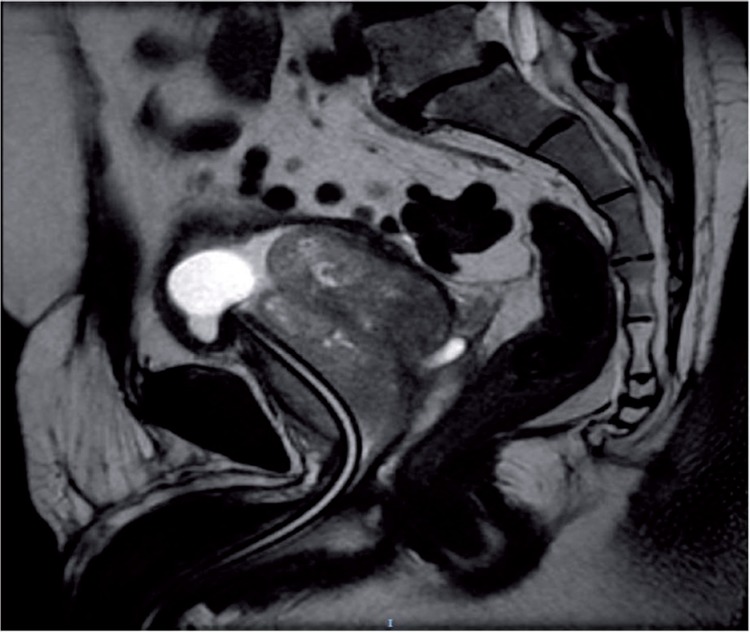
T2-weighted prostate MRI in sagittal view showing a diffusely enlarged prostate. Note also the indwelling bladder catheter displaced anteriorly.

**Figure 2:**
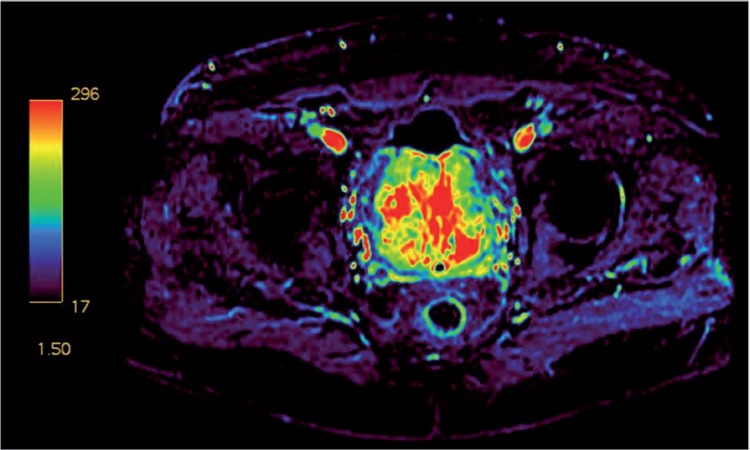
Prostate MRI contrast-enhanced perfusion map showing a markedly increased perfusion.

**Figure 3:**
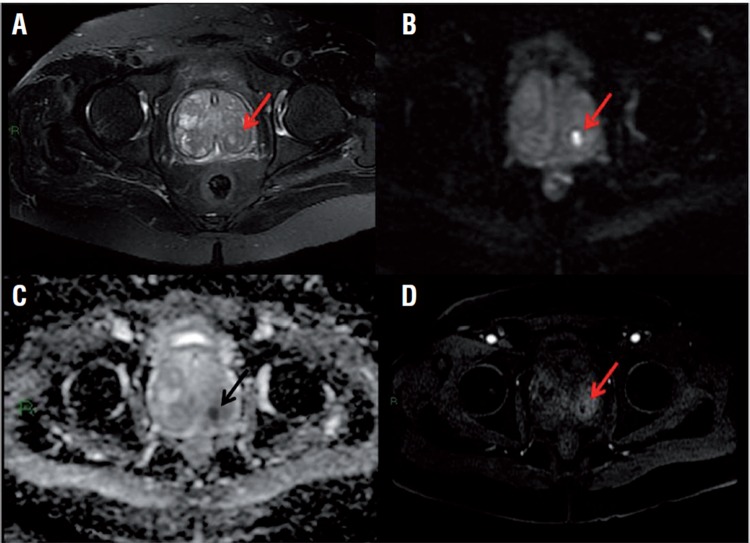
Prostate MRI showing a small nodular area (arrows) in the left posterior mid-third of the transition zone. This lesion shows liquid content on T2-weighted images (A), and marked diffusion restriction (B), with corresponding ADC map (C) and intense peripheral enhancement (D), findings suggestive of an abscess.

PSA levels were 6.54ng/mL and digital rectal examination showed a diffusely enlarged prostate without focal nodulations. He was submitted to prostatic multiparametric MRI to exclude a concomitant neoplasia that demonstrated an enlarged prostate (Figure-1) with increased vascularization on perfusion map (Figure-2). A small nodule of abnormal diffusion restriction in the left posterior mid-third of the transition zone, with intense peripheral post-contrast enhancement and a liquefied center was also identified, suggestive of a microabscess (Figure-3). Despite the focal lesion, final PI-RADS score was 2 (a score used to predict the risk of malignancy on multiparametric MRI-(1)), indicating low probability of a significant prostatic neoplasia. Ultrasound-guided biopsy was performed after multiparametric MRI due to PSA levels, including a targeted biopsy on the area described.

The histopathological analysis of the fragments on the targeted area (suggestive of abscess) evidenced a chronic granulomatous inflammatory process and the specific test for acid-alcohol resistant bacilli (BAAR) confirmed mycobacterial etiology.

## DISCUSSION

Tuberculous prostatitis is a rare granulomatous prostate disease that predominantly occurs in immune-compromised patients and may be mistaken for prostate cancer on clinical, laboratory and imaging findings (2-7). Genitourinary tuberculosis accounts for 10% of cases of extrapulmonary tuberculosis, and like other sites of genitourinary involvement, symptoms of prostatic tuberculosis are nonspecific. In two case series (5, 7), the most common presentation was irritative voiding symptoms followed by hemospermia. Sterile pyuria may also be a suggestive clinical feature (2-4). In those series, none of the patients presented respiratory symptoms and only one patient had suggestive findings of previous tuberculosis on chest x-ray (5, 7). Tuberculous prostatitis affects most commonly middle-aged man and may elevate PSA levels and alter digital rectal examination (enlarged prostate with hard consistency, eventually, with firm nodulations), characteristics shared with prostate cancer (2-4).

Imaging examinations may help in diagnostic elucidation, but there is also overlap with features of prostatic adenocarcinoma. A series of cases (6) suggested two imaging patterns on MRI: a nodular pattern, represented by several small nodules in the peripheral zone, usually markedly hypointense on T2-weighted images and no significant diffusion restriction and a diffuse pattern, in which the whole peripheral zone is affected by hypointense bands in T2, but less evident when compared to the nodular pattern (5, 6).

The presence of a prostatic abscesses, as in the present case, is an infrequent but characteristic finding and appears on MRI as a nodular area of intense diffusion restriction and peripheral enhancement (5, 6).

Since there may be an overlap of clinical, laboratory and imaging findings, the gold standard for diagnosis is histopathology, confirming caseous granulomas like any other site of tuberculosis infection, as well as the presence of BAARs when stained by the Ziehl-Neelsen method. Treatment usually involves the use of classic anti-tuberculosis drugs (2-4).
